# Measuring irregular migration in Chile: The use of data linkage methods

**DOI:** 10.23889/ijpds.v8i2.2188

**Published:** 2023-09-14

**Authors:** Belén Harnecker, Felipe Mallea

**Affiliations:** 1 National Migration Service, Santiago, Chile

Foreign population in Chile almost doubled between 2017 and 2021. In recent years, the sources to measure the phenomenon has been the 2017 Census, plus administrative data from the migratory institutions. Yet, traditional estimation methods have not included irregular migration in all its forms, despite the fact that between 2018 and 2022 illegal entries grew by 754%. Official numbers had only included (1) people registered by the day of the census, and (2) people that, from that day at least applied for one residence permit. However, records from other public institutions (education, health and police) suggests there are a considerable number of migrants that meets neither of the two criteria, that don’t have and official Chilean ID (RUN), but have registered in different services with provisional identifiers.

In order to properly account for irregular migration flows, this exploratory study presents a method of deterministic integration of personal administrative records from different public institutions (police, health, education and migrations), of foreign people that don’t have a unique identifier (RUN), using personal variables: names, surnames and date of birth. The proposed methodology can be summarized in four steps: (1) cleaning and standardizing records and variables (2) building an identification key and removing exact duplicated records in each data set (3) removing partial duplicated records in each data set using fuzzy matching techniques, and (4) matching the data sets using fuzzing matching techniques. The result of this procedure is the number of foreign people that showed signs of being residents by registering in the public services, but don’t have any registration of visa applications with the National Migration Service. These individuals, according with the existing law (Law N° 21.325), live in a (probable) irregular status.

This study not only represents and innovation in estimating foreign population in Chile as it accounts for inhabitants in irregular situation, but it also proposes a novel standard of registration and integration of personal administrative records of different public services.

